# Prevalence and associations of neurodevelopmental and mental health disorders with academic and well-being challenges among nursing and midwifery students: A cross-sectional study

**DOI:** 10.18332/ejm/195808

**Published:** 2024-12-12

**Authors:** Alina Liepinaitiene, Vaidas Jotautis, Simona Jankauskaite, Lijana Navickiene, Daiva Bartusiene, Evelina Lamsodiene, Rasa Gaiziunaite, Vaida Valalyte, Rasa Vaitkiene, Magdalena Korżyńska-Piętas, Linda Gudre, Viktorija Piscalkiene

**Affiliations:** 1 Kauno kolegija Higher Education Institution, Kaunas, Lithuania; 2 Vytautas Magnus University, Faculty of Natural Sciences, Department of Environmental Sciences, Kaunas, Lithuania; 3 Medical University of Lublin, Lublin, Poland; 4 Ryga First Medical College of the University of Latvia, Ryga, Latvia

**Keywords:** midwifery students, nursing students, mental health disorders, prevalence, well-being challenges

## Abstract

**INTRODUCTION:**

This study highlights the significant stress faced by nursing and midwifery students stemming from the rigorous requirements of their curriculum, which includes both demanding theoretical and practical elements. This study aims to identify the causes and manifestations of learning environment stress among midwifery and nursing students.

**METHODS:**

A cross-sectional study in Lithuania, Poland and Latvia was employed to evaluate students’ learning challenges and neurodiversity using 40 different questions assessed on a Likert scale. Data were gathered through online surveys in February–April 2024, distributed to nursing and midwifery students from three international institutions.

**RESULTS:**

The findings reveal significant correlations (p<0.05) between anxiety and stress levels in students with neurodiversity, underscoring the critical impact of stress on the mental health and well-being of nursing students. These results demonstrate significant associations (ρ=0.2; p<0.05) with shifts in life meaning, health issues, emotional and cognitive challenges, and mobbing.

**CONCLUSIONS:**

Anxiety and stress are significantly related to the presence of neurodisability among students, highlighting the necessity for targeted mental health interventions to address these critical issues.

## INTRODUCTION

Studying nursing and midwifery is among the most demanding emotionally and physically fields in the higher education system. Nursing and midwifery students are under unique pressure because their curriculum requires skills that directly affect the health and lives of patients. In addition, they face various challenges during their studies, which include both theoretical and practical aspects. For these reasons, nursing and midwifery students often experience high levels of stress, which can have a negative impact on their academic performance, psychological well-being, and overall health^1,2^.

The nursing and midwifery degree programs require intensive theoretical and practical training. Theoretical training covers a wide range of medical knowledge, from anatomy and physiology to pharmacology and pathology. These take a lot of time and effort to understand and master. In addition, practical training includes clinical sessions where students work directly with patients and apply their knowledge to real situations. This practical aspect can be particularly stressful, as students often feel underprepared or afraid of making a mistake that could have serious consequences for patients^2,3^.

One of the main stressors is academic demands. Studying nursing and midwifery is intensive, and students often have many exams, assignments, and projects. These assignments require constant preparation and learning, and students often feel overwhelmed. In addition, nursing and midwifery degree programs often require high academic standards, so students feel constant pressure to achieve the highest results^4^.

Another important stress factor is clinical training. During clinical practice, students have to work in hospitals or other healthcare facilities, where they come into contact with patients and their problems. This experience can be stressful, as students often feel underprepared or afraid of making mistakes. In addition, in clinical practice, they may encounter difficult or even traumatic situations that may have a long-term effect on their psychological state^5,6^.

In addition to academic and clinical demands, nursing and midwifery students also face a variety of personal challenges. Many students have to juggle their studies with work, family commitments, and other responsibilities. These can cause additional stress and difficulties in balancing studies and personal life. In addition, social support relationships with friends and family can significantly impact student stress. Lack of support or conflict can leave students feeling isolated and powerless^7^.

Finally, cultural and social factors can also influence stress. Students from different cultures may face additional challenges in understanding and adapting to cultural differences. They may feel lonely or discriminated against, which adds to the stress. Social norms and expectations can also affect students’ psychological state, especially if they cannot meet societal or family expectations^3^.

Given the factors listed above, it is important to understand that stress in nursing and midwifery students is a complex and multifaceted problem. Helping students overcome these challenges requires developing support systems and strategies to help them manage stress and maintain good psychological and physical health. This can include individual and institutional support mechanisms such as counseling, mentoring, healthcare services, and stress management training. This is the only way to ensure that nursing and midwifery students can graduate and become competent and caring healthcare professionals^6,8^.

Healthcare education is uniquely demanding. Students in these fields must engage in rigorous academic coursework while undergoing intense clinical training. This dual burden can lead to heightened levels of stress and anxiety as students struggle to balance theoretical knowledge with practical skills. Time pressures, high expectations, and the emotional toll of dealing with real patients can exacerbate feelings of inadequacy, fear of failure, and burnout. These stressors are not merely transient; they can have lasting effects on mental health, leading to conditions such as generalized anxiety disorder, depression, and burnout syndrome^9^.

Research indicates that mental health issues are disturbingly prevalent among university students in general, but they are particularly pronounced among those pursuing healthcare professions. For example, systematic reviews have noted that nursing and midwifery students often report higher levels of anxiety and depression compared to their peers in other disciplines. This raises urgent questions about how educational institutions support their students’ mental health and how these issues may impact the quality of care provided to patients in the future^10^.

In addition to general mental health concerns, attention deficit hyperactivity disorder (ADHD) presents additional challenges for students in academic settings. ADHD can significantly impair concentration, organizational skills, and time management – crucial for success in healthcare education. Students with ADHD may experience difficulties in absorbing large volumes of information, meeting deadlines, and performing under pressure, further contributing to stress and anxiety levels. Educational institutions need to recognize these unique challenges and provide tailored support systems that can help ADHD-affected students thrive academically while maintaining their mental well-being^11^.

Therefore, this study aims to determine the causes and expression of learning environmental stress of midwifery and nursing students.

A secondary aim is to investigate associations between the aforementioned neurodevelopmental and mental health disorders and various challenges impacting students’ well-being and academic performance, including specific learning difficulties, changes in the meaning of life, health challenges, emotional-cognitive difficulties, and mobbing, among the two groups of students: nurses and midwives.

## METHODS

### Study design and participations

A cross-sectional study evaluated students’ learning difficulties using 40 questions, assessed on a 5-point Likert scale ranging from 1 (lowest agreement) to 5 (highest agreement). Four developmental and mental health disabilities attention deficit hyperactivity disorder (ADHD), stress, depression, and anxiety) were presented to gauge neurodiversity expression, with students having options to self-identify or diagnose. Learning achievements were assessed based on the last semester’s average.

The survey encompassed students from Kauno kolegija Higher Education Institution (hereafter referred to as Kauno kolegija HEI), Lublin Medical University (Poland), and Ryga 1st Medical College of Latvia University (Latvia), all specializing in nursing and midwifery. An online questionnaire link was distributed to all Kauno kolegija HEI students, providing survey details and emphasizing the importance of their participation. The survey was conducted from February to May 2024 and utilized an online platform across institutions in three different countries.

In total, 147 students participated. Table 1 provides a comprehensive breakdown of the student population based on three primary categories: country of origin, gender, and study program.

**Table 1:** Sociodemographic characteristics of nursing and midwifery students who participated in the study (N=147)

*Characteristics*	*n*	*%*	*Percent of total number of midwifery and nursing students in institution*
**Country**			
Lithuania	103	70.1	26.7
Poland	37	25.1	34.2
Latvia	7	4.8	29.2
**Gender**			
Female	140	95.2	
Male	5	3.4	
Do not want to reveal	2	1.4	
**Study program**			
Midwifery	79	53.7	
Nursing	68	46.3	

We describe the findings of research conducted on nursing students between February and April 2024, focusing on the difficulties they faced and the correlations with ADHD, depression, anxiety, and stress. A total of 68 nursing students participated in the study. The correlations between these factors and their significance levels provide insight into the relationships between various difficulties and mental health issues. We also describe the findings of research conducted on midwifery students between February and April 2024, focusing on their difficulties and the correlations with ADHD, depression, anxiety, and stress. The study involved 79 midwifery students, and the correlations between these factors are presented with their respective significance levels.

Analyzing results by study program rather than country is statistically ethical since this approach ensures a more accurate, relevant, and fair comparison of educational outcomes. Study programs offer a standardized framework, minimizing the variability introduced by differing national educational standards and practices. This homogeneity within programs allows for meaningful conclusions that directly reflect the educational quality and effectiveness of specific curricula and teaching methods. Institutions can target improvements more effectively by focusing on study programs, ultimately leading to enhanced educational practices and student outcomes. This method respects the integrity of the data and ensures that comparisons are made consistently and equitably, thereby upholding statistical and ethical standards.

### Ethical approval and informed consent

The approval of the Kauno kolegija HEI Applied Research Ethics Compliance Review Committee was obtained for the study (Protocol No. 13-14 of 29 January 2024). The research adhered to fundamental ethical guidelines. Participants had the option to decline involvement. Adults who willingly consented completed a questionnaire for the study. Beforehand, respondents were provided with details regarding the objectives of the research, its nature, and the opportunity to review the findings. They were assured that their anonymity and confidentiality would be maintained, with no collection of personal information (such as name, surname, or personal identification number), and that the study findings would be presented in an aggregated format.

### Statistical analysis

The statistical analysis was conducted utilizing SPSS 23 and Microsoft Office software. Normal distribution was verified using skewness and kurtosis measures. Spearman’s correlation coefficient (ρ) evaluated the relationship strength and nature between variables. At the same time, ANOVA tested for statistically significant differences between means of independent group parameters, considering a p<0.05 indicative of a significant relationship.

Factor analysis, employing the Kaiser-Meyer-Olkin (KMO) measure of sampling adequacy (0.88), approximated chi-squared (χ^2^=8491.07) and degrees of freedom (df=780) with a significance level (Sig.=0.000), and was employed to condense variables into factors representing shared characteristics. This analysis, employing VARIMAX rotation of principal components, revealed five factors, the majority of which met the condition L ≥0.6. Cronbach’s alpha assessed the reliability of the questionnaire on learning difficulties, demonstrating high internal consistency across the five scales (40 items total) with a Cronbach’s alpha of 0.95.

## RESULTS

In the category of country of origin, the data reveal that students from Lithuania make up the largest group, with 103 students accounting for 70.1% of the total in this category and 26.7% of the total student population at the institution (Table 1). Polish students follow, with 37 representing 25.1% of the country-specific category and 34.2% of the overall student body. Students from Latvia form the smallest group, with seven students making up 4.8% of the country-specific category and 29.2% of the institution’s total student population.

Regarding gender distribution, the student population is predominantly female, with 140 female students constituting 95.2%. Male students are significantly fewer, with only five students making up 3.4%. Additionally, two students chose not to reveal their gender, accounting for 1.4% of the student population.

Regarding the study programs, participants were divided into two groups. There are 79 students enrolled in the Midwifery program, making up 53.7% of the total number of students in this category. The Nursing program has 68 students, representing 46.3% of the category.

This detailed demographic breakdown highlights the diversity and composition of the student population within the institution, offering insights into the predominant nationalities, gender distribution, and chosen fields of study among the students. The exact sociodemographic information of students is presented in Table 1.

Table 2 presents data on the prevalence of various disabilities suspected among students, categorized by study program – Nursing and Midwifery. The disabilities include attention deficit hyperactivity disorder (ADHD), depression, anxiety, and stress, with each category further divided into three subcategories: those who do not have the disability, those who suspect they have the disability themselves, and those whose disability has been diagnosed. All difficulties scales came from the main results and factorial analysis.

**Table 2:** Distribution of nursing and midwifery students who participated in the study, by neurodiversity (N=147)

*Disabilities suspected of /by students*	*Do not have*		*Suspected by themselves*		*Identified and approved*		*Sig.*
	*n*	*%*	*n*	*%*	*n*	*%*	
**Attention deficit hyperactivity disorder (ADHD)**							0.053
Nursing	36	24.5	26	17.7	6	4.0	
Midwifery	44	29.9	33	22.5	2	1.4	
**Depression**							
Nursing	31	20.1	33	22.5	4	2.7	0.076
Midwifery	23	15.6	50	34.0	6	4.1	
**Anxiety**							
Nursing	24	16.3	38	25.9	6	4.0	**0.004**
Midwifery	14	9.5	59	40.1	6	4.0	
**Stress**							
Nursing	10	6.8	49	33.3	9	6.1	**0.003**
Midwifery	16	10.9	63	42.9	0	0.0	

For ADHD, in the Nursing program, 36 students (24.5%) do not have this syndrome, 26 students (17.7%) suspect they may have it, and six students (4.0%) have had it identified and approved by doctors. In the Midwifery program, 44 students (29.9%) identified as not having ADHD, 33 students (22.5%) suspect they have it, and two students (1.4%) have had it identified and approved by doctors. The statistical level of significance (Sig.) for ADHD was 0.053 (ρ=0.425).

For depression, 31 nursing students identified (20.1%) as not having depression, 33 students (22.5%) identified as suspect they have it, and four students (2.7%) have had it identified and approved by doctors. In the Midwifery program, 23 students (15.6%) do not have depression, 50 students (34.0%) suspect they have it, and six students (4.1%) have had it identified and approved by doctors. The statistical level of significance was 0.076 (ρ=0.625).

Regarding anxiety, 24 nursing students identified (16.3%) as not having anxiety, 38 students (25.9%) identified as suspect they have it, and six students (4.0%) have had it identified and approved. In the Midwifery program, 14 students (9.5%) identified as not having anxiety, 59 students (40.1%) identified as suspect they have it, and six students (4.0%) have had it identified and approved by doctors. The statistical level of significance for anxiety was 0.004 (ρ=0.421).

For stress, 10 nursing students (6.8%) identified as not having stress, 49 students (33.3%) identified that they suspect they have it, and nine students (6.1%) have had it identified and approved. In the Midwifery program, 16 students (10.9%) identified as not having stress, 63 students (42.9%) identified that they suspect they have it, and none had it identified and approved by doctors. The statistical level of significance for stress was 0.003 (ρ=0.128).

These data provide insights into the self-reported and confirmed prevalence of mental health disabilities among students in different study programs, highlighting the levels of suspected and confirmed cases within the Nursing and Midwifery student sample. Both anxiety and stress show significant correlations with neurodisabilities among students (p=0.004 and p=0.003, respectively). These results suggest that higher levels of anxiety and stress are significantly associated with the presence of neurodisabilities within the student.

Specific learning difficulties showed negative correlations with ADHD (ρ= -0.23, p=0.129), depression (ρ= -0.22, p=0.161), anxiety (ρ= -0.23, p=0.131), and stress (ρ= -0.10, p=0.216). However, these correlations were not statistically significant, as all significance levels were above 0.05 (Table 3).

**Table 3:** Difficulties and correlation with ADHD, depression, anxiety and stress of midwifery students who participated in the study (N=79)

*Scales of difficulties*	*ADHD*	*Depression*	*Anxiety*	*Stress*
	*ρ Sig.*	*ρ Sig.*	*ρ Sig.*	*ρ Sig.*
Specific learning difficulties	-0.232	-0.215	-0.231	-0.103
	0.129	0.161	0.131	0.216
Change in meaning of life	0.152	0.193	0.235	**0.297**
	0.325	0.210	0.125	**0.000**
Health challenges	0.124	**0.0397**	**0.435**	**0.380**
	0.422	**0.008**	**0.003**	**0.000**
Emotional-cognitive difficulties	1.082	0.219	**0.309**	**0.339**
	0.599	0.153	**0.041**	**0.000**
Mobbing	0.201	-0.011	0.038	**0.295**
	0.191	0.944	0.805	**0.000**

Changes in the meaning of life were positively correlated with ADHD (ρ=0.15, p=0.325), depression (ρ=0.19, p=0.210), anxiety (ρ=0.24, p=0.125), and stress (ρ=0.3, p=0.000). Among these, only the correlation with stress was statistically significant, with a significant level of 0.000.

Health challenges showed significant positive correlations with depression (ρ=0.4, p=0.008), anxiety (ρ=0.44, p=0.003), and stress (ρ=0.38, p=0.000), while the correlation with ADHD (ρ=0.12, p=0.422) was not significant. These results suggest significant relationships between health challenges and the mental health factors of depression, anxiety, and stress.

Emotional-cognitive difficulties were positively correlated with anxiety (ρ=0.31, p=0.041) and stress (ρ=0.34, p=0.000), with both correlations being statistically significant. The correlations with ADHD (ρ=1.08, p=0.599) and depression (ρ=0.22, p=0.153) were not significant.

Mobbing had a positive but non-significant correlation with ADHD (ρ=0.20, p=0.191), a very weak and non-significant negative correlation with depression (ρ= -0.01, p=0.944), a non-significant positive correlation with anxiety (ρ=0.04, p=0.805), and a significant positive correlation with stress (ρ=0.3, p=0.000).

It highlights the significant correlations between various factors affecting midwifery students, particularly the strong associations between stress and changes in the meaning of life, health challenges, emotional-cognitive difficulties, and mobbing. The significance levels indicate that stress is a critical factor correlated with students’ multiple challenges.

Specific learning difficulties had negative correlations with ADHD (ρ= -0.16, p=0.294), depression (ρ= -0.23, p=0.137), anxiety (ρ= -0.26, p=0.085), and stress (ρ= -0.07, p=0.432). However, none of these correlations was statistically significant, as all significance levels exceeded the threshold of 0.05 (Table 4).

**Table 4:** Difficulties and correlation with ADHD, depression, anxiety and stress of nursing students who participated in the study (N=68)

*Scales of difficulties*	*ADHD*	*Depression*	*Anxiety*	*Stress*
	*ρ Sig.*	*ρ Sig.*	*ρ Sig.*	*ρ Sig.*
Specific learning difficulties	-0.162	-0.228	-0.263	-0.065
	0.294	0.137	0.085	0.0432
Change in meaning of life	0.157	0.122	0.193	**0.298**
	0.308	0.432	0.208	**0.000**
Health challenges	0.134	**0.329**	**0.424**	**0.380**
	0.384	**0.029**	**0.004**	**0.000**
Emotional-cognitive difficulties	0.077	0.145	0.255	**0.334**
	0.619	0.348	0.094	**0.000**
Mobbing	0.224	-0.030	0.044	**0.255**
	0.143	0.844	0.778	**0.002**

Changes in the meaning of life were positively correlated with ADHD (ρ=0.16, p=0.308), depression (ρ=0.12, p=0.432), anxiety (ρ=0.19, p=0.208), and stress (ρ=0.3, p=0.000). Among these, only the correlation with stress was statistically significant, with a significant level of 0.000.

Health challenges showed significant positive correlations with depression (ρ=0.33, p=0.029), anxiety (ρ=0.42, p=0.004), and stress (ρ=0.38, p=0.000). The correlation with ADHD (ρ=0.13, p=0.384) was not significant.

Emotional-cognitive difficulties were positively correlated with ADHD (ρ=0.08, p=0.619), depression (ρ=0.15, p=0.348), anxiety (ρ=0.26, p=0.094), and stress (ρ=0.33, p=0.000). Only the correlation with stress was statistically significant, indicating a significant relationship between emotional-cognitive difficulties and stress.

Mobbing exhibited a positive correlation with ADHD (ρ=0.22, p=0.143), a very weak negative correlation with depression (ρ= -0.03, p=0.844), a weak positive correlation with anxiety (ρ=0.04, p=0.778), and a significant positive correlation with stress (ρ=0.26, p=0.002).

Overall, the research highlights significant correlations between stress and various difficulties nursing students face, including changes in the meaning of life, health challenges, emotional-cognitive difficulties, and mobbing. These significant relationships underscore stress’s critical role in nursing students’ mental health and well-being.

## DISCUSSION

This study presents critical insights into the prevalence and correlations of various mental health disabilities among nursing and midwifery students. The data highlight significant associations, particularly involving stress and anxiety, and underscore the need for targeted mental health interventions in these student populations.

The prevalence of ADHD among nursing and midwifery students was notable, although the difference between the two groups was not statistically significant. This finding suggests that ADHD is a common concern in both programs, reflecting the broader literature that emphasizes the impact of ADHD on students in rigorous academic settings^12^. Prior studies have shown that ADHD can significantly affect academic performance and stress levels, necessitating adequate support and accommodations for affected students^13^.

Depression was prevalent among the students, with a substantial proportion suspecting they had a disability. However, the difference in depression rates between nursing and midwifery students was not significant. This result aligns with previous research indicating high rates of depressive symptoms among healthcare students due to their demanding academic and clinical environments^14^. These findings suggest that mental health support should be integral to healthcare education programs to help mitigate these challenges.

Anxiety was significantly more prevalent among midwifery students compared to nursing students. This higher prevalence may be attributed to midwifery students’ unique stressors, such as their emotional intensity and high responsibility in clinical placements. This observation is consistent with existing literature that identifies higher anxiety levels in midwifery students due to the nature of their clinical duties^15^.

The study found that stress levels were significantly higher among midwifery students than nursing students. Stress was significantly correlated with several challenges, including changes in the meaning of life, health challenges, emotional-cognitive difficulties, and mobbing. These correlations highlight the pervasive impact of stress on students’ well-being, reinforcing findings from other studies that stress is a critical factor affecting mental health in healthcare education^16^. Stress management interventions are crucial to help students cope with these pressures.

The significant correlations between stress and various difficulties faced by students emphasize the critical role of stress in influencing mental health. For example, stress was significantly correlated with changes in the meaning of life, health challenges, and emotional-cognitive difficulties. These findings are supported by other studies that show stress negatively impacts students’ mental health and academic performance, exacerbating emotional and cognitive challenges^17^.

The significant correlation between stress and mobbing highlights the detrimental effects of bullying and harassment on students’ mental health. Previous research has also found that exposure to mobbing significantly increases stress levels, leading to severe psychological distress^18^. Addressing these issues is vital for creating a supportive and safe learning environment for students.

### Limitations

Students’ self-identification determined neurodiversity, which may have been influenced by social desirability bias or a lack of self-awareness, potentially leading to inaccurate or biased responses regarding their neurodiversity and learning challenges. Secondly, the survey was conducted solely at one higher education institution in Lithuania, one in Latvia, and one in Poland. The small number of respondents may limit the possibility of general conclusions or generalizations.

## CONCLUSIONS

This study underscores the critical role of stress and anxiety in contributing to mental health disabilities among nursing and midwifery students. The significant correlations between these factors and various mental health challenges highlight the need for targeted interventions to support students’ mental health. Educational institutions must have mental health resources and create supportive environments to mitigate these stressors and enhance students’ well-being.

## Data Availability

The data supporting this research are available from the authors on reasonable request.
